# Palivizumab for Donor‐Derived RSV Infection in an Adult Bilateral Lung Transplant Recipient: A Case Report

**DOI:** 10.1002/rcr2.70706

**Published:** 2026-07-29

**Authors:** Rosemary Meharry, Cassandra Vale, John Mackintosh

**Affiliations:** ^1^ Western General Hospital Edinburgh UK; ^2^ The Prince Charles Hospital Intensive Care Unit Brisbane Australia; ^3^ The Prince Charles Hospital Queensland Lung Transplant Service Brisbane Australia; ^4^ Faculty of Medicine University of Queensland Brisbane Australia

**Keywords:** lung, palivizumab, RSV, transplant

## Abstract

Respiratory syncytial virus (RSV) causes significant morbidity in immunocompromised adults. We describe a post‐operative bilateral lung transplant recipient with donor‐derived RSV who developed increasing viral load and progressive respiratory failure despite conventional therapy (enteral ribavirin, methylprednisolone, IVIG) and was ultimately administered palivizumab. By day 42, the patient remained ventilator‐dependent with low RSV PCR cycle threshold (Ct) values on bronchoalveolar lavage. Transbronchial biopsies showed mixed inflammatory appearances, indicating either acute RSV infection or possible rejection. Following administration of single dose intramuscular palivizumab (10 mg/kg) we observed an improving clinical trajectory and increasing Ct values. RSV became undetectable by day 64, with follow‐up biopsies revealing no rejection. The patient was successfully discharged on day 87 and continues to show stable allograft function. This case demonstrates safe use of single dose palivizumab to augment conventional therapy for life‐threatening RSV in the profoundly immunocompromised lung transplant recipient.

## Graphical abstract

We describe the use of off‐label palivizumab in an early post‐operative bilateral lung transplant recipient with probable donor‐derived RSV who developed persistent severe LRTI and respiratory failure. RSV PCR Ct fell and remained low from day 0 to day 42 despite conventional therapy until palivizumab was administered on day 44, following which we observed an improving RSV PCR Ct and sustained clinical recovery. RSV PCR became negative 20 days post palivizumab.

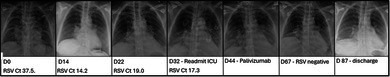

## 1 Introduction

Respiratory Syncytial Virus (RSV) is a recognised cause of morbidity in immunocompromised adults and in the lung transplant population is variably implicated in the development of acute and chronic lung allograft dysfunction (CLAD) [1]. With limited evidence base, usual therapeutic approaches include intravenous immunoglobulin (IVIG), ribavirin and corticosteroids as well as modifying background immunosuppression. Vaccine‐mediated prevention is effective, but cost and access have prevented widespread uptake amongst potential candidates. Limited literature on monoclonal antibody therapy for RSV post‐transplant is largely based on haematopoietic stem cell transplant recipients or late stage (> 3 months post) solid organ transplantation (SOT) experiences. We describe the use of off‐label palivizumab in an early post‐operative bilateral lung transplant recipient with donor‐derived RSV refractory to conventional therapy.

## 2 Case Report

A 58‐year‐old female with short telomere syndrome (telomere length < 10th centile, likely pathogenic TERT variant) associated with idiopathic pulmonary fibrosis (IPF) tested positive for RSV on day 0 following bilateral lung transplantation. She received usual induction with basiliximab, tacrolimus (CNI), mycophenolate mofetil (MMF) and methylprednisolone before continuing standard maintenance immunosuppression with corticosteroids (CS) + CNI + MMF. As she was highly sensitised (PRA 95%) with a positive virtual crossmatch at transplant, IVIG 2 g/kg was added.

**FIGURE 1:**
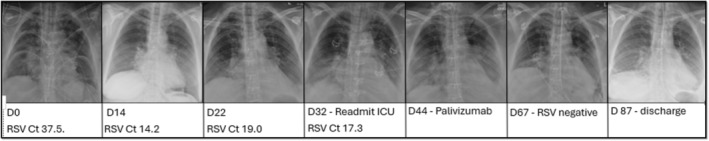
Chest radiograph series documenting radiological changes with concurrent RSV cycle threshold (Ct) values and clinical course. Serial chest x‐rays displaying progressive radiological consolidation from Day 0 (D0) until readmission to ICU on Day 32 (D32) alongside contemporaneous quantitative viral loads (RSV Ct values). Following Palivizumab administration on Day 44 (D44) subsequent images demonstrate improving pulmonary infiltrates with corresponding rising Ct and viral clearance by Day 67 (D67). Radiological appearances on day of discharge (D87) shown.

**FIGURE 2:**
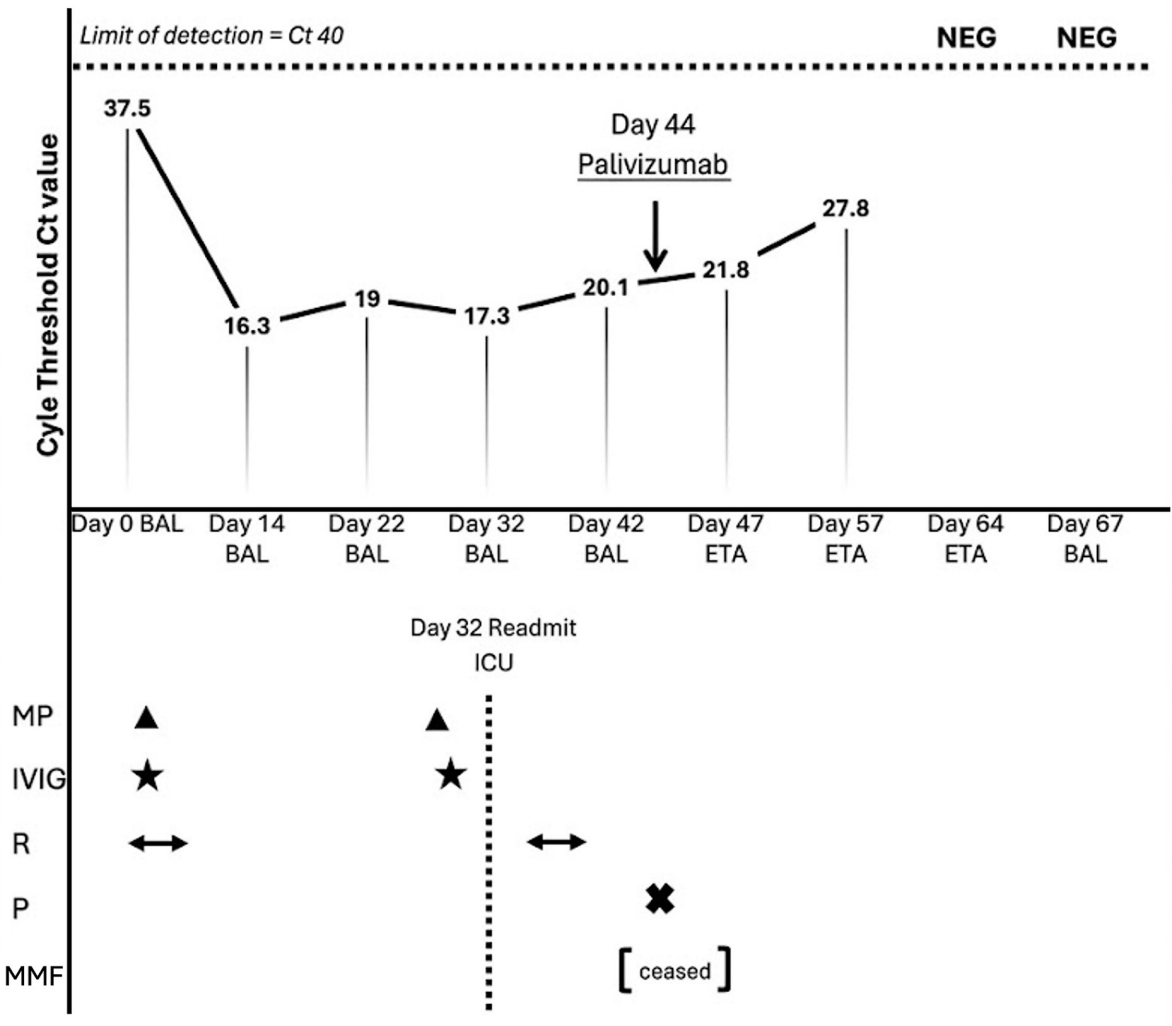
Low viral PCR cycle threshold (Ct) indicative of higher concentration of viral genetic material, associated with increased infectivity. High Ct indicates low concentration of viral genetic material. Ct becomes negative when it rises to > 40. BAL, bronchoalveolar lavage; ETA, endotracheal aspirate. Conventional therapy: Intravenous immunoglobulin (IVIG) on Day 1 and 30. PO ribavirin (R) Days 1–5 and Days 36–40. Methylprednisolone (MP) was pulsed at 250 mg 8 hourly on induction/reperfusion and a further methylprednisolone pulse was given on Day 29. Palivizumab (P) Day 44. Mycophenolate mofetil (MMF) was ceased Day 42–47. Readmission to ICU on Day 32 denoted by dashed line.

RSV was detected with a PCR cycle threshold (Ct) approaching the limit of detection on bronchoalveolar lavage (BAL) obtained on admission to ICU following transplant. We suspected donor‐derived RSV and enteral ribavirin was prescribed. Initially the patient recovered as expected: extubated on Day 2 and stepped down from ICU on Day 8. However, promising early progress was complicated by progressive respiratory failure with reducing RSV PCR Ct on subsequent BALs suggesting a high viral load (nadir RSV Ct 16.3 Day 14, Figure 2). This correlated with worsening dyspnoea, wheeze and squeaks on auscultation, progressive radiological consolidation (Figure 1) and hypercapnoeic respiratory failure precipitating re‐intubation and readmission to ICU on Day 32.

Transbronchial biopsies demonstrated mixed appearances potentially reflecting A1‐2 rejection, aspiration or acute RSV; no other pathogens were isolated. Given ongoing RSV PCR positivity with low Ct (17.3), a second course of enteral ribavirin was prescribed alongside high dose methylprednisolone. MMF was temporarily withheld before being recommenced at a reduced dose due to leucopoenia.

At Day 42 the patient remained ventilator dependent with RSV pneumonia (Ct 20.1). We elected to trial off‐label, single dose palivizumab 10 mg/kg (800 mg) intramuscularly on Day 44 post‐transplant. Dosing was extrapolated from the limited literature available reporting palivizumab use in adult SOT [2, 3, 4].

Following palivizumab administration, we observed improvements in both RSV PCR Ct and respiratory symptoms (Day 47 Ct 21.8, rising to 27.8 by Day 57—Figure 2). During this period, our patient was able to engage in rehabilitation, wean ventilator settings and progress toward extubation. RSV PCR became undetectable (Ct > 40) 20 days post palivizumab (Day 64), with repeat biopsies at Day 67 showing resolution of inflammatory appearances and absence of rejection. The patient left ICU on Day 77, was discharged on Day 87 and post‐discharge, allograft function continued to improve. Surveillance biopsies (Days 107 and 191) showed no evidence of rejection, and the patient's functional recovery is sustained through 12 months' follow‐up.

## 3 Discussion

Lung transplantation carries an inherent risk of donor‐derived infection to the potential detriment of recipient outcomes. Real world data [4] shows Nirsevimab, an RSV‐specific monoclonal antibody, is effective for RSV prevention in infants but clinical trials for use in adult SOT are lacking. Existing data on Palivizumab for RSV in SOT is limited, often involving late post‐transplant administration and variable dosing (7.5–15 mg/kg). In the largest multicentre study of RSV outcomes in lung transplant, median time to infection was 28.7 months and only 3 cases of 77 received Palivizumab [5]. A further study of a multidrug regimen (including IV palivizumab) treating 26 SOT RSV cases had a median time to infection of 845 days with no progression to RSV LRTI and sustained graft function through 6 months of follow up [6]. Our experience expands the evidence suggesting that palivizumab is not only a viable adjunctive therapy for refractory RSV in SOT but is also safe with no noted toxicity in the early post operative window.

This case underscores the necessity of pre‐transplant RSV vaccination, acknowledging that the true incidence and impact of donor‐derived RSV infection is unknown. Although the RSV vaccine is recommended to all transplant candidates assessed by our unit, this is currently off‐licence, presenting a significant financial barrier often preclusive to patient uptake. Clinical trials continue to evaluate the immunogenicity and safety of RSV in adult SOT (NCT06067230). It remains to be seen whether a fully subsidised RSV vaccination program for potential recipients and the post lung transplant population will reduce morbidity and mortality in this vulnerable group.

Some confounding factors should be considered in this case. Due to leucopoenia, MMF was temporarily ceased Days 42–47, which may have contributed to viral clearance. Also, it should be noted that little is known of the comparability of PCR Ct from differing clinical specimens. We elected to obtain lower airway specimens by endotracheal aspirates (ETA) as a minimally invasive comparable alternative to repeated, potentially high risk BALs—an approach corroborated by one prior study correlating ETA and BAL Ct for pneumocystis jiroveci pneumonia in ICU patients.

Acknowledging these complexities, the patient nonetheless demonstrated a consistently rising RSV PCR Ct following Palivizumab administration (Figure 2), suggesting reducing viral load which correlated with sustained clinical recovery. The patient's allograft function continues to increment in keeping with ongoing recovery from a protracted ICU admission. Now 1 year post‐transplant, she demonstrates improving performance status and remains under close assessment. The long‐term effects of early, severe RSV infection from an allograft function perspective are yet to be determined.

In summary, this case demonstrates safe use of single dose palivizumab to augment conventional therapy in early and life‐threatening, refractory RSV infection in the profoundly immunocompromised lung transplant recipient. We acknowledge that this single case does not attest to the efficacy of palivizumab in this context and more robust evidence is required to validate a standardised RSV prevention and treatment regimen—including any role for monoclonal antibody therapy—in the post lung transplant population.

## Author Contributions

All listed authors contributed substantially to the conception and drafting of this case study as well as undertaking critical review and agreeing the final approved version submitted for publishing.

## Funding

The authors have nothing to report.

## Ethics Statement

The authors declare full compliance with national regulations in the off‐label use of palivizumab in this case. Our patient's elected power of attorney gave informed written consent prior to administration of off‐label palivizumab.

## Consent

The authors declare that written informed consent was obtained for the publication of this manuscript and accompanying images and attest that the form used to obtain consent from the patient complies with the Journal requirements as outlined in the author guidelines.

## Conflicts of Interest

The authors declare no conflicts of interest.

## Data Availability

The data that support the findings of this study are available on request from the corresponding author. The data are not publicly available due to privacy or ethical restrictions.
